# Improved apple latent spherical virus-induced gene silencing in multiple soybean genotypes through direct inoculation of agro-infiltrated *Nicotiana benthamiana* extract

**DOI:** 10.1186/s13007-018-0286-7

**Published:** 2018-03-06

**Authors:** C. R. Gedling, E. M. Ali, A. Gunadi, J. J. Finer, K. Xie, Y. Liu, N. Yoshikawa, F. Qu, A. E. Dorrance

**Affiliations:** 1 Department of Plant Pathology, The Ohio State University, 1680 Madison Avenue, Wooster, OH 44691 USA; 2 Department of Horticulture and Crop Science, The Ohio State University, 1680 Madison Avenue, Wooster, OH 44691 USA; 3 School of Chemistry and Biological Engineering, University of Science and Technology Beijing, Beijing, China; 4 MOE Key Laboratory of Bioinformatics, Center for Plant Biology, Tsinghua-Peking Joint Center for Life Sciences, School of Life Sciences, Tsinghua University, Beijing, 100084 China; 5 Plant Pathology Lab, Facility of Agriculture, Iwate University, Morioka, Japan; 6 Present Address: Washington State University, 1100 N Western Ave., Wenatchee, WA 98801 USA

**Keywords:** Apple latent spherical virus, VIGS, Soybean functional analysis

## Abstract

**Background:**

Virus induced gene silencing (VIGS) is a powerful genomics tool for interrogating the function of plant genes. Unfortunately, VIGS vectors often produce disease symptoms that interfere with the silencing phenotypes of target genes, or are frequently ineffective in certain plant genotypes or tissue types. This is especially true in crop plants like soybean [*Glycine max* (L.) Merr]. To address these shortcomings, we modified the inoculation procedure of a VIGS vector based on *Apple latent spherical virus* (ALSV). The efficacy of this new procedure was assessed in 19 soybean genotypes using a soybean *Phytoene desaturase* (*GmPDS1*) gene as the VIGS target. Silencing of *GmPDS1* was easily scored as photo-bleached leaves and/or stems.

**Results:**

In this report, the ALSV VIGS vector was modified by mobilizing ALSV cDNAs into a binary vector compatible with *Agrobacterium tumefaciens*-mediated delivery, so that VIGS-triggering ALSV variants could be propagated in agro-infiltrated *Nicotiana benthamiana* leaves. Homogenate of these *N. benthamiana* leaves was then applied directly onto the unifoliate of young soybean seedlings to initiate systemic gene silencing. This rapid inoculation method bypassed the need for a particle bombardment apparatus. Among the 19 soybean genotypes evaluated with this new method, photo-bleaching indicative of *GmPDS1* silencing was observed in nine, with two exhibiting photo-bleaching in 100% of the inoculated individuals. ALSV RNA was detected in pods, embryos, stems, leaves, and roots in symptomatic plants of four genotypes.

**Conclusions:**

This modified protocol allowed for inoculation of soybean plants via simple mechanical rubbing with the homogenate of *N. benthamiana* leaves agro-infiltrated with ALSV VIGS constructs. More importantly, inoculated plants showed no apparent virus disease symptoms which could otherwise interfere with VIGS phenotypes. This streamlined procedure expanded this functional genomics tool to nine soybean genotypes.

**Electronic supplementary material:**

The online version of this article (10.1186/s13007-018-0286-7) contains supplementary material, which is available to authorized users.

## Background

Virus-induced gene silencing (VIGS) down-regulates the expression of a targeted plant gene following inoculation of the plants with a recombinant virus vector that carries a portion of the coding sequence of this gene [1–5]. This approach exploits the RNA silencing-mediated antiviral defense in plants, which uses double-stranded RNA (dsRNA) of virus origin as a template to produce small interfering RNAs (siRNAs) that in turn target single-stranded viral RNAs for degradation in a sequence-specific manner [1–5]. Thus, plant gene fragments inserted in the virus genome become a source of siRNAs, which then target the corresponding plant gene mRNA for destruction, causing VIGS [5].

Since its inception, many VIGS vectors including *Tobacco rattle virus* (TRV) [6–8], *Potato virus X* (PVX) [9] and *Tomato golden mosaic virus* (TGMV) [10] have enabled silencing of numerous genes in different plant hosts. Although many VIGS vectors have since been developed for various crop plants including maize (*Zea mays*) [11], soybean [4, 12, 13], and wheat (*Triticum aestivum*) [12, 13], these vectors often require extensive and lengthy delivery procedures and can result in variable rates of silencing. For instance, the most widely used VIGS vector for soybean, based on the *Bean pod mottle virus* (BPMV), requires a modified BPMV cDNA to be delivered into soybean leaves or lima bean (*Phaseolus lunatus*) cotyledons using particle bombardment to amplify the inoculum [3, 14–17]. Another limitation of the BPMV and other VIGS systems are the foliar symptoms that develop due to virus infection such as chlorosis, necrosis, and leaf distortion. These symptoms can interfere with the observation of phenotypic changes caused by silencing of plant genes. More importantly, some VIGS vectors are reported to have limited movement within the leaf and stem tissue, resulting in uneven phenotypes in different plant tissues [18].

Our goal was to identify a VIGS vector that can be used to assess functions of soybean genes active in specific tissue types, such as roots and seeds. The *Apple latent spherical virus* (ALSV)-based VIGS system was chosen for testing and further modifications because it was previously reported to be an effective, versatile VIGS tool in more than 20 plant species including soybean [19]. ALSV was first isolated from apple (*Malus pumila*) in Japan and has a wide host range including cucurbits, *Nicotiana* spp., *Rosaceae* fruit trees and legumes [19, 20]. ALSV is a member of the genus *Cheravirus,* family *Secoviridae* [21]. Its genome consists of two single-stranded, positive sense RNA (RNA1 and RNA2), with RNA1 encoding all proteins essential for genome replication and RNA2 encoding the movement protein and three capsid proteins referred to as VP25, VP20, and VP24 [21, 22].

ALSV has been used to silence soybean genes and found to be capable of silencing genes in developing soybean seeds [23, 24]. In a previous report, use of ALSV vector in soybean involved a multi-step inoculum propagation and delivery procedure, which evaluated few soybean cultivars (Enrei, Dewamusume, Tanbaguro, Suzukari, Chamame) [23]. In the first study, *GmPDS1* was used as a target of ALSV VIGS [23] and propagated in *Chenopodium quinoa*. Those symptomatic leaves were then homogenized and used to re-inoculate *C. quinoa* plants in order to amplify the modified virus. ALSV viral RNA were then extracted from the second batch of infected *C. quinoa*, and used to inoculate germinating soybean seeds via particle bombardment [19, 23, 24]. We initially sought to reproduce the reported results through a similar inoculation procedure, using the soybean cultivar Jack reported in the study of Yamagishi and Yoshikawa [23], as well as two other soybean genotypes, Wyandot and PI 567301B. In this current study, the ALSV vector was mobilized into a binary vector that permits its delivery into *Nicotiana benthamiana* leaves via agro-infiltration. A more streamlined inoculation procedure, using infected *N. benthamiana* homogenate to directly rub-inoculate the first unifoliate of young soybean seedlings, was tested on 19 soybean genotypes. This new method allowed for inoculum propagation within 5–10 days and eliminated the need for particle bombardment. This method also allowed us to identify nine additional soybean genotypes that showed a substantial level of susceptibility to ALSV-mediated VIGS.

## Methods

### Modification of ALSV VIGS vector system and generation of VIGS constructs

**Fig. 1:**
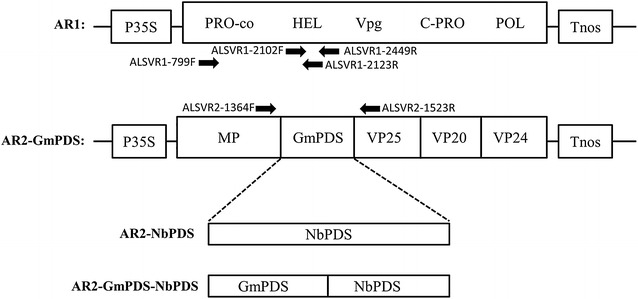
Schematic representation of ALSV RNA1 (AR1), as well as three modified forms of ALSV RNA2 (AR2): AR2-GmPDS, AR2-NbPDS, and AR2-GmPDS-NbPDS, all of them flanked by a 35S promoter (P35S) and a NOS terminator (Tnos) to facilitate the synthesis of primary transcripts by RNA polymerase II of the host plant. Arrows correspond to primers used to amplify the corresponding regions of ALSV. GmPDS and NbPDS correspond to fragments derived from *GmPDS1* and *NbPDS* coding sequences. A target gene can be inserted between MP and VP25 using *Xho*I and *Bam*HI restriction enzymes

The original ALSV VIGS vector plasmids, pEALSR1 (ALSV-RNA1; 6813 nt) and pEALSR2L5R5 (ALSV-RNA2; 3385 nt), were constructed in Dr. Yoshikawa’s lab at Iwate University, as previously described [19]. To facilitate agro-infiltration delivery, the ALSV cDNA inserts in these two plasmids were migrated into the binary vector pBinPlusARS to create pYL-AR1 and pYL-AR2L5R5, respectively, in Dr. Yule Liu’s lab. The pYL-AR2L5R5 has two unique restriction enzyme sites, *Bam*HI and *Xho*I, that can be used for inserting nonviral fragments. Three different VIGS constructs were generated, targeting *N. benthamiana Phytoene desaturase* (*NbPDS*), *GmPDS1* of soybean, or both (Fig. 1). pYL-ALSV-RNA2-NbPDS (simplified as AR2-NbPDS hereafter) contained an NbPDS fragment that is identical to the one used in [19]. AR2-GmPDS contained a 327-nt fragment of *GmPDS1* coding sequence, corresponding to nt #1521-1847 of NM_001249840. It differs from the one used in previous studies [19, 23] in that the new *GmPDS1* insert is slightly longer (327 vs. 300 nucleotides), and corresponds to a different region of *GmPDS1*. Nevertheless, this 327 bp fragment has been shown to induce robust *GmPDS1* silencing when it was inserted into a BPMV-based VIGS vector [3]. Additionally, this *GmPDS1* fragment was inserted into the ALSV-RNA2 (AR2) cDNA vector in an inverted orientation, with all in-frame stop codons eliminated by point mutations, to improve silencing efficiency [3]. Finally, AR2-GmPDS-NbPDS contained both of the above mentioned inserts in tandem (Fig. 1). All inserts were synthesized as gBlocks fragments by IDT (Coralville, IA), and cloned into the *Bam*HI-*Xho*I-digested pYL-AR2L5R5 using Gibson Assembly (NEB, Ipswich, MA). The inserts in all constructs were sequenced to confirm their identities.

### Transformation of *A. tumefaciens* with the VIGS constructs, and inoculation of *N. benthamiana* plants via agro-infiltration

The ALSV-derived binary constructs, namely pYL-AR1, AR2-GmPDS, AR2-NbPDS, and AR2-GmPDS-NbPDS, were transformed into *Agrobacterium tumefaciens* strain C58C1 [25] with electroporation and transferred to selective Luria–Bertani media (LB: 0.17 M NaCl, 0.5% yeast extract, 1.0% tryptone, 1.5% agar) containing three antibiotics: kanamycin (50 μg/ml), rifampicin (50 μg/ml), and gentamycin (50 μg/ml) [25]. Colony PCR was carried out on the resulting *A. tumefaciens* colonies using appropriate primers to confirm the transformation (Additional file 1). To prepare for agro-infiltration of *N. benthamiana*, the transformed *A. tumefaciens* strains were grown by transferring single colonies into culture tubes containing 3 ml LB with the same antibiotics, and incubated on a shaker overnight at 28 °C. These cultures were then further diluted 1:100 and shaken for another 18 h. The *Agrobacterium* was then pelleted by centrifuging at 4000 rpm for 20 min, and re-suspended in agro-infiltration buffer (10 mM MgCl_2_, 10 mM MES pH 5.7, 100 µM acetosyringone), and the concentration determined by measuring OD600 values. All agro-suspensions were diluted to OD600 = 1. Each agro-suspension containing the AR2 derivatives (AR2-GmPDS, AR2-NbPDS, and AR2-GmPDS-NbPDS) were mixed with pYL-AR1 in order to initiate ALSV infections in infiltrated plant leaves. Another agro-suspension containing a plasmid expressing the p19 silencing suppressor was also included in every agro mixture [26].

The *N. benthamiana* plants used for infiltration were grown in a mixture of sterile soil and potting mix (Miracle Gro, Scotts Co. LLC, Marysville, OH) in the greenhouse. The *Agrobacterium* mixtures were then infiltrated into the first two true leaves of 3 weeks old *N. benthamiana* plants using 3 ml needless syringes (Becton–Dickinson, Franklin Lakes, NJ). Infiltrated plants were kept in the dark overnight, and then moved into a growth chamber (CMP6010 Adaptis, Conviron, Winnipeg, MB, Canada) on a 12/12 h day/night with 24/22 °C day/night [25]. Infected tissue of *N. benthamiana* leaves was collected 5–10 days after infiltration (dai). The homogenate consisting of infiltrated *N. benthamiana* leaves and inoculation buffer was rub-inoculated on the first true leaves of soybean seedlings. Inoculated soybean plants were kept in the dark for 24 h, and then moved into a growth room at room temperature (20–25 °C).

### RNA extraction

RNA was extracted from young infected *N. benthamiana* and *C. quinoa* leaves using a RNeasy kit (Qiagen, Hilden, Germany) following the manufacturers protocol. RNA quality was assessed following gel electrophoresis of a 1% agarose gel, and the concentration was measured using a Nanodrop 1000 spectrophotometer (Nanodrop Technologies, Wilmington, DE). RNA concentration was adjusted to 300–500 ng/µl for particle bombardment experiments.

### Particle bombardment inoculation of soybean seedlings

For particle bombardment inoculations, the procedure reported in [23] was followed, with minor modifications to accommodate bombardment using particle inflow gun [16, 27, 28]. For this purpose, ALSV VIGS constructs were first propagated in agro-infiltrated *N. benthamiana* leaves, followed by another round of amplification in *C. quinoa* plants. Total RNA was then extracted from *C. quinoa* leaves. Upon confirmation of the ALSV RNA using RT-PCR, the total RNA was then precipitated onto tungsten particles, and used to bombard germinating soybean embryos at 2.5–5 µg per bombardment, following procedure in [23]. Soybean embryos were bombarded at a distance of 10, 13 and 14 from the bombardment filter of the particle inflow gun, using 50 PSI of pressurized helium gas. These experiments were repeated three times.

### Reverse transcriptase PCR

The presence of the ALSV in *N. benthamiana* as well as *C. quinoa* leaves was confirmed using reverse transcriptase polymerase chain reaction (RT-PCR). One µg of RNA was DNAse treated (Invitrogen, Carlsbad, CA) and then reverse transcribed using a qScript cDNA synthase kit (Quanta BioSciences, Gaithersburg, MD) and 10 pmol of oligo(dT) following the manufacturer’s protocol, with the following specific primer pairs: ALSVR1-2102F and ALSVR1-2449R, ALSVR1-799F and ALSVR1-2123R, or ALSVR2-1364F and ALSVR2-1523R (Additional file 1). The PCR conditions were 94 °C for 2 min, 32 cycles of 94 °C for 30 s, 55 °C for 30 s, 72 °C for 1 min, and followed by a final extension at 72 °C for 5 min. Amplified products were visualized on 1% agarose gel by gel electrophoresis for validation of amplicon presence and size.

### Semi-quantitative PCR

Semi-quantitative PCR was used to quantify the amount of PDS transcript in inoculated soybean leaves, and the presence of ALSV-RNA1 (AR1; Additional file 1). PCR was carried out on cDNA using primers GmPDS-938F and GmPDS-1464R, to quantify PDS transcript levels and ALSV-R1-2102F and ALSV-R1-2449R to quantify AR1 levels using GoTaq Flexi DNA polymerase (Promega, Madison, WI). Actin was used as a control and was amplified using primers GmACT101-479F and GmACT101-579R [29]. Both reactions were performed using 50 ng of cDNA, 2.5 mM dNTP, and 100 pmol of primers using the following conditions: 94 °C for 2 min, then 32 cycles of 94 °C for 30 s at, 57 °C for 30 s at, and 72 °C for 1 min., with a final extension at 72 °C for 5 min. Annealing temperature was adjusted depending on the primer pair used. Amplified products were visualized after electrophoresis on a 1% agarose gel.

### Sensitivity to ALSV VIGS among 19 soybean genotypes

There were 19 soybean genotypes (Table 1) selected for evaluation of susceptibility to ALSV VIGS which included plant introductions and adapted cultivars. For these studies, four seeds of each genotype were planted in 10 cm pots in a mix of sterile soil and potting mix (Miracle Gro, Scotts Co. LLC, Marysville, OH), which were then placed in the greenhouse at 25 °C under a 14 h photoperiod, watered twice daily with no fertilization or insecticide spray.

**Table 1:** Nineteen soybean genotypes silencing efficiency of GmPDS-VIGS silencing through the means of rub-inoculation method

Genotype	# of plants showing viral symptoms^a^/# inoculated	# of plants showing photo-bleaching^a^/# inoculated	% Efficiency^a^
Jack	3/8	1/8	12.5
Wyandot	0/8	0/8	0
Williams	4/8	0/8	0
Williams 82	4/8	0/8	0
Thorne	0/8	0/8	0
PI 24350	2/8	3/8	37
NIL 2640	0/8	0/8	0
L83-570	0/8	0/8	0
OX-20	0/8	0/8	0
Conrad	4/8	0/8	0
Sloan	3/8	0/8	0
Magellan	3/8	3/8	38
PI 427106	7/7	2/7	29
PI 399073	10/16	10/16	63
PI 424354	11/15	7/15	47
PI 427105B	2/8	0/8	0
PI 567516C	12/15	9/15	60
PI 567301B	24/24	24/24	100
RIL 301	24/24	24/24	100

ALSV virus infection and *GmPDS1* silencing efficiency of 19 genotypes, plant introductions, public cultivars, and germplasm by rub-inoculations. Symptoms were observed at soybean growth stage V2 approximately 21 dai with GmPDS-VIGS. Data as a summary of 2–3 replicates overtime with 4 plants in each replicate

^a^The total rating across multiple plants and replicates

Soybeans were rub-inoculated 2 weeks after planting on the first unifoliate leaves. Each unifoliate was inoculated with the homogenate of the *N. benthamiana* leaves that were agro-infiltrated with ALSV VIGS constructs. Inoculated plants were placed in the dark for 24 h, then transferred to a growth chamber at 22 °C under a 12/12 h day/night photoperiod and watered twice a day with deionized water by misting to increase humidity. Three weeks after inoculation, leaf tissue was collected and total RNA was extracted. This experiment was repeated three times with 4 plants per genotype in each experiment.

### Apical puncture inoculation of soybean seedlings

A subset of three soybean genotypes was also inoculated with a procedure referred to as apical puncture in an attempt to deliver ALSV VIGS constructs directly into meristematic tissues. Seeds of cultivars Thorne, Wyandot, and Jack were first germinated for 2–3 days in a mixture of sterile soil and potting mix in a 10 cm pot. Two to three days after germination, when cotyledons emerged but before the first unifoliate leaf appeared, a small incision was made ~ 5–10 mm in length on the proximal half of one of the two cotyledons. The shoot apex was exposed and punctured with a dissecting needle 6–8 times, and 50 µl of ground *N. benthamania* infected homogenate and inoculation buffer was pipetted onto the wound. Control plants where inoculated with 50 µl of inoculation buffer.

## Results

### Particle bombardment delivery of ALSV-based VIGS vector with a *GmPDS1*-targeting insert yielded low rate of ALSV infection in soybean

**Fig. 2:**
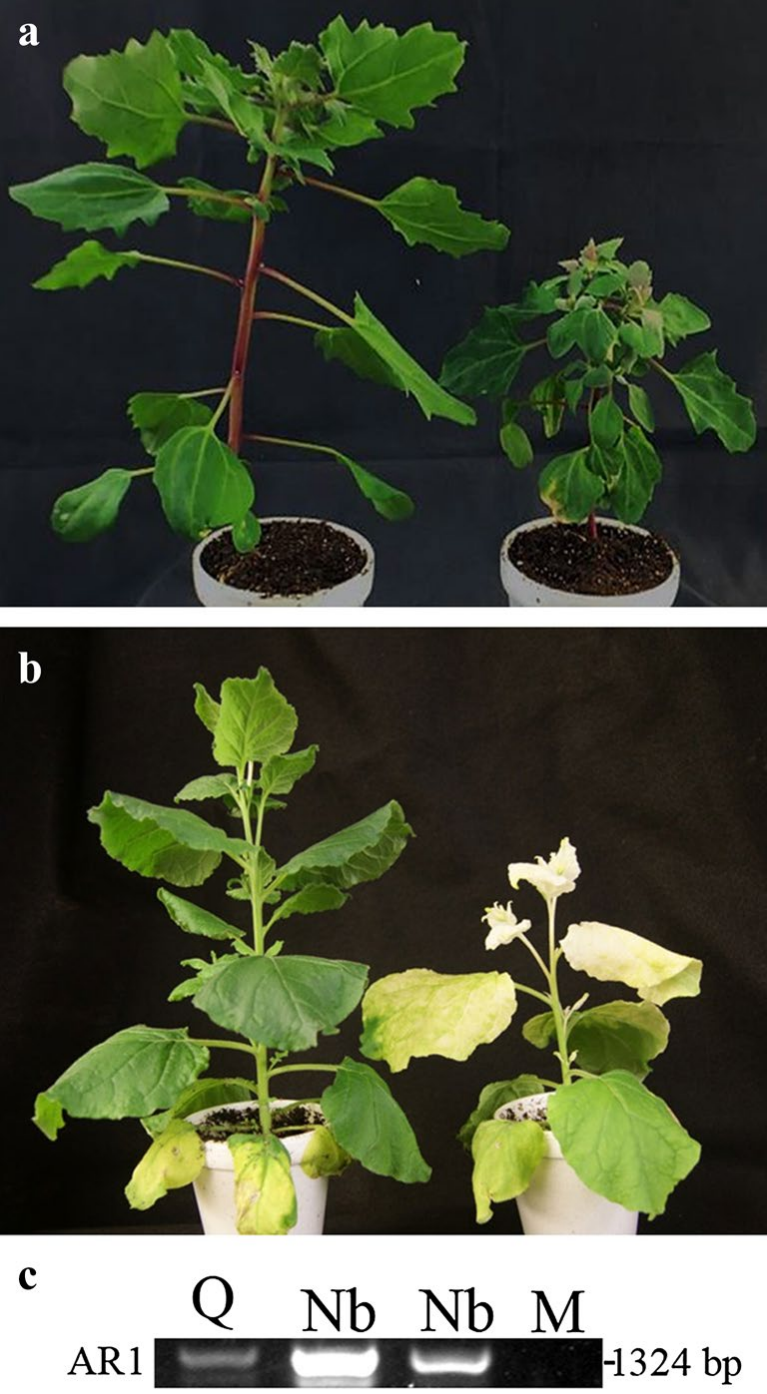
Successful infection of *Chenopodium quinoa* and *Nicotiana benthamiana* plants by ALSV derivatives. **a** *C. quinoa* plants infected with inoculation buffer only (mock inoculated, left) or ALSV containing a modified RNA2 (AR2-GmPDS) (right). **b** *N. benthamiana* infiltrated with buffer (left), or Agrobacterium suspensions containing AR1, AR2-NbPDS, and p19 (right). **c** RT-PCR detection of AR1 in systemic leaves of two *C. quinoa plants* inoculated with AR1 plus AR2-NbPDS (Q, Q), and two *N. benthamiana* plants (Nb, Nb) infiltrated with AR1, AR2-NbPDS, and p19. Mocks are *N. benthamiana* (M1) and *C. quinoa* (M2) plants treated with inoculation buffer

We first attempted to reproduce the particle bombardment approach reported in [23]. To do this, the AR2-GmPDS construct, along with another plasmid harboring AR1 cDNA (pEALSR1) [23], was used to propagate viral RNA in *C. quinoa*, followed by introduction of viral RNA into germinating soybean seeds. Successful infection of *C. quinoa* was confirmed by viral symptoms of stunting as well as RT-PCR detection of ALSV RNAs (Fig. 2a, c). Despite repeated attempts with strict adherence to the published procedures [16, 17, 28], we failed to observe any photo-bleaching or symptoms relating to silencing of *GmPDS1* in the plants that emerged from the bombarded seeds, or detect ALSV RNAs in the plant tissue using RT-PCR (data not shown).

### A modified set of ALSV vectors permit the propagation of VIGS constructs in *N. benthamiana* via agro-infiltration

The propagation of VIGS inoculum with agro-infiltrated *N. benthamiana* leaves and subsequent inoculation of soybean seedlings with *N. benthamiana* extracts were then evaluated [30]. ALSV infects *N. benthamiana* [19, 31], a plant species which is also highly susceptible to agro-infiltration mediated DNA delivery. We reasoned that migrating the ALSV cDNAs into a binary vector destined for *Agrobacterium* should allow us to deliver these cDNAs into *N. benthamiana* cells via agro-infiltration, and propagate the VIGS-mediating ALSV derivatives from modified cDNAs in *N. benthamiana* leaves. The cDNAs of both AR1 and modified AR2, along with the flanking 35S promoter and NOS terminator, were excised from pEALSR1 and pEALSR2L5R5 and incorporated into the binary vector pBinPlusARS to yield pYL-AR1 and pYL-AR2L5R5, respectively.

To ensure the new set of vectors initiate sufficiently robust infections in *N. benthamiana*, we developed AR2-NbPDS which would induce photo-bleaching in *N. benthamiana* if successfully propagated. *Agrobacterium* suspensions harboring AR2-NbPDS and AR1, as well as another construct expressing the p19 silencing suppressor of *Tomato bushy stunt virus*, were mixed and delivered into *N. benthamiana* leaves with agro-infiltration [25, 26, 32]. Successfully infected plants were confirmed through RT-PCR (Fig. 2c). Photo-bleached upper non-inoculated leaves were visible at 10 days after infiltration (10 dai), and symptoms expanded to almost all of the young leaves by 14 dai (Fig. 2b). Notably, infiltrated leaves harvested from these plants at 5 dai, as well as photo-bleached leaves harvested up to 25 dai, when homogenized and used as inoculum to inoculate new plants via rub-inoculation, induced the same levels of photo-bleaching as agro-infiltration (data not shown). Therefore, at least in *N. benthamiana* plants, the agro-infiltrated leaves contained ALSV derivatives at levels sufficient to induce silencing via rub-inoculation in secondary plants. These results suggest that this ALSV propagation procedure is very effective for amplifying ALSV silencing constructs.

### ALSV VIGS constructs propagated in *N. benthamiana* induce robust silencing in soybean

**Fig. 3:**
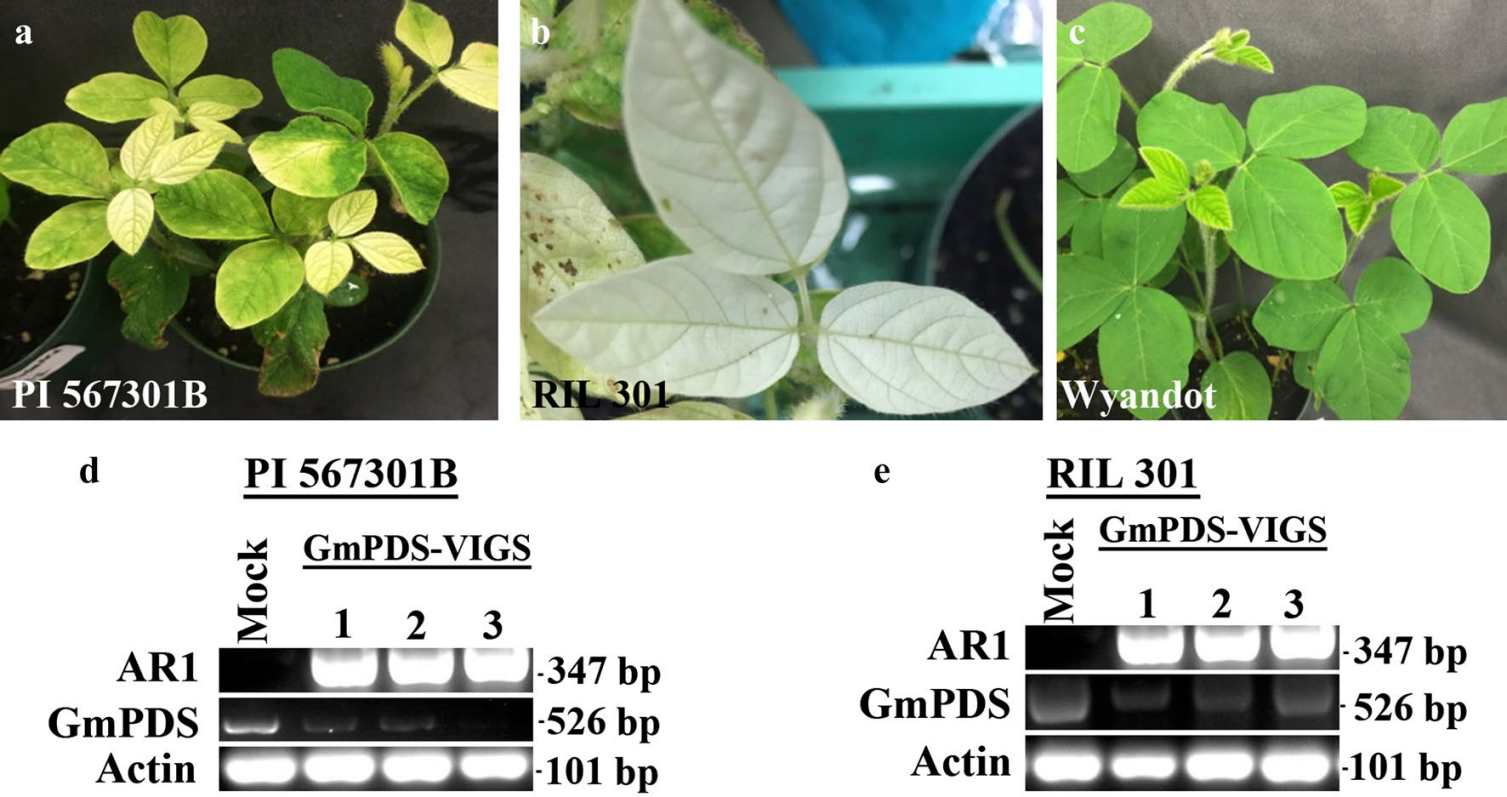
Different soybean genotypes inoculated with extracts of *N. benthamiana* leaves that were agro-infiltrated with AR1, AR2-GmPDS, and p19 **a** PI 567301B, **b** RIL 301, **c** Wyandot, **d** semi-quantitative RT-PCR with RNA extracted from three symptomatic systemic leaves from three separate plants of PI 567301B (**d**), and RIL 301 (**e**)

Having established that ALSV VIGS derivatives propagated in *N. benthamiana* leaves readily induced target gene silencing of young (3 weeks old) *N. benthamiana* plants, we then evaluated their efficacy for inducing silencing in soybean. First, we amplified the *GmPDS1*-targeting ALSV derivative by infiltrating *N. benthamiana* leaves with mixed *Agrobacterium* suspensions containing the pYL-AR1, AR2-GmPDS, and p19 constructs. The homogenate of 5 dai infiltrated leaves was applied to the unifoliate leaves of three different soybean genotypes, Wyandot, PI 567301B, and RIL 301 (a recombinant inbreed line derived from a cross of Wyandot x PI 567301B), at 10–12 days after germination. Photo-bleaching as an indication of *GmPDS1* silencing was observed in of PI 567301B (Fig. 3a) and RIL 301 (Fig. 3b) genotypes at 14 and 21 days post inoculation (dpi), respectively. By 30 dpi, 100% of PI 567301B and RIL 301 plants showed extensive photo-bleaching that spread to majority of the young and systemic leaves. However, no photo-bleaching was observed in Wyandot plants (Fig. 3c). We also confirmed the presence of AR1 in the systemically infected leaves in the silenced soybean genotypes with RT-PCR (Fig. 3d, e). The reduction of *GmPDS1* RNA levels was only detected in PI 567301B and RIL 301 plants, as expected from the photo-bleaching phenotype. This experiment was repeated 3 times with consistent results (Table 1). Together these results indicate that agro-infiltrated *N. benthamiana* leaves are a reliable source of ALSV inoculum for soybean VIGS. They also suggest that different soybean genotypes may respond to ALSV-administered VIGS with different levels of susceptibility.

### Soybean genotypes differentially respond to ALSV-mediated VIGS

**Fig. 4:**
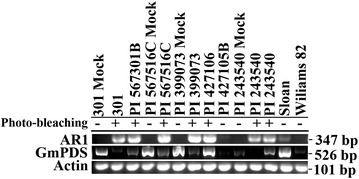
Detection of AR1 and *GmPDS1* transcripts in different soybean genotypes with semi-quantitative RT-PCR on genotypes of systemic leaves. Plus sign indicates photo-bleaching symptoms were observed, minus sign indicates no photo-bleaching symptoms. The soybean actin mRNA was used as the control to ensure a similar amount of RNA was used in all lanes. Top—AR1, middle—*GmPDS1*, bottom—actin

Having observed the robust *GmPDS1* silencing in PI 567301B and RIL 301 but not in Wyandot, the other parent of RIL 301, we assessed the effectiveness of the ALSV-GmPDS VIGS vector in 16 additional soybean genotypes, using the *GmPDS*1-targeting inoculum prepared in *N. benthamiana*. Nine genotypes responded to ALSV-mediated *GmPDS1* silencing (Table 1) with visible leaf wrinkling. Consistent with earlier results, all PI 567301B and RIL 301 plants developed extensive photo-bleaching. Three genotypes (PI 399073, PI 567516C, and PI 424354) yielded silencing in 40–63% plants. Three additional genotypes (Magellan, PI 24350, and PI 427106) exhibited 29–39% infection and silencing. The cultivar Jack, which was also included in the previous study [23], only exhibited 12.5% efficiency in this set of experiments. However, in subsequent experiments described later, we observed consistently higher rates of photo-bleaching in Jack that allowed for additional experiments testing photo-bleaching throughout different developmental stages. In contrast, ten remaining genotypes, including the model cultivar, Williams 82, did not develop symptoms of visible *GmPDS1* silencing even though the ALSV RNA was detected albeit at different concentrations in several of the inoculated genotypes (Fig. 4). These results clearly demonstrate that the effectiveness of the ALSV-mediated VIGS as described in this study for soybean is genotype-dependent.

### Apical puncture inoculation

**Fig. 5:**
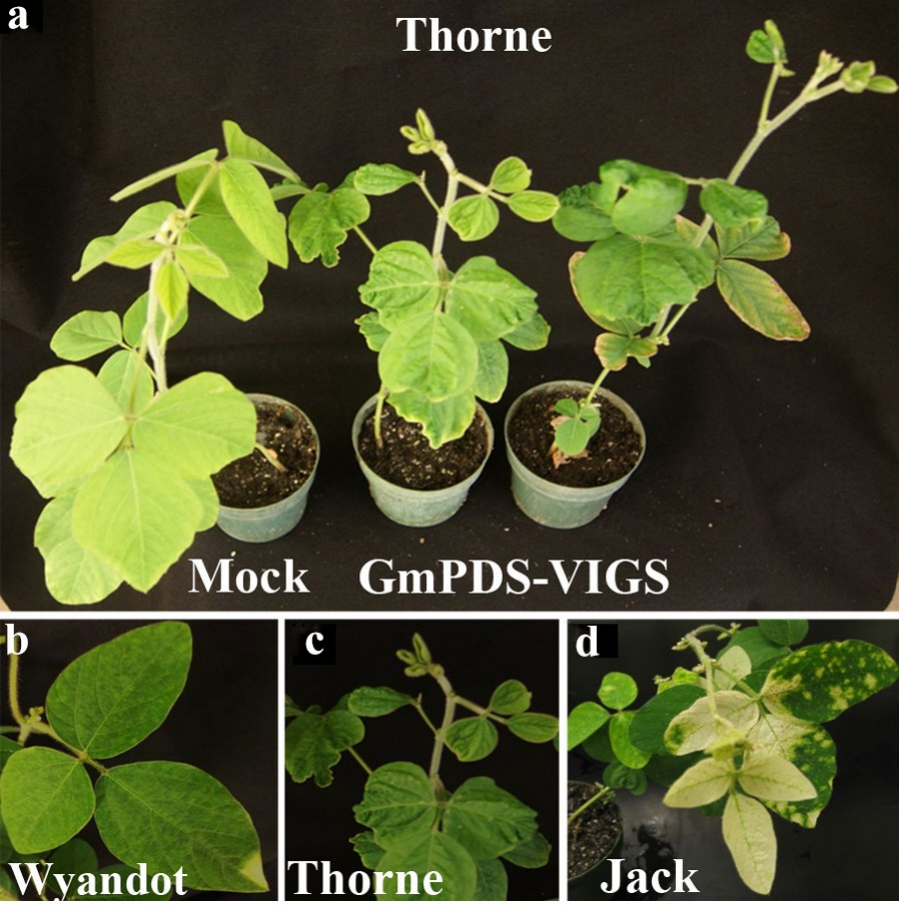
Apical puncture inoculation of *N. benthamiana*-propagated, *GmPDS1*-targeting VIGS inoculum on three soybean genotypes, all images were taken at 21 dai. **a** Cultivar Thorne inoculated buffer (mock, left), or *GmPDS1* VIGS-triggering inoculum (two plants on the right). **b**–**d** Wyandot, Thorne, and Jack plants inoculated with the same GmPDS1 VIGS-triggering inoculum

A subset of three genotypes, Jack, Thorne, and Wyandot, that showed low (12.5%) to no response to GmPDS-VIGS with rub-inoculation were also inoculated using a novel approach referred to as apical puncture in an attempt to reach apical meristem of young seedlings (see Methods for details). With this inoculation procedure, ~ 70% of plants from all three genotypes exhibited symptoms of ALSV infection. However, while 60% of Jack plants developed visible photo-bleaching, only one leaf of one Wyandot plant showed photo-bleaching spots. None of the Thorne plants showed any signs of photo-bleaching (Fig. 5, Table 2). Thus, the apical puncture inoculation procedure improved *GmPDS1* silencing in the Jack cultivar, but not in Wyandot or Thorne cultivars.

**Table 2:** Soybean genotypes GmPDS-VIGS efficiency using the apical puncture inoculation method

Genotype	# of plants showing viral symptoms^a^/# inoculated	Plant (%) showing Photo-bleaching^a^/# inoculated	% Efficiency^a^
Thorne	35/50	0/50	0
Wyandot	1/50	0/50	0
Jack	30/50	30/50	60

Soybean genotypes inoculated through the means of apical puncture. Symptoms observed after 21 dai. Percentage of plants showing symptoms was calculated by dividing the number of plants with symptoms divided by the total number of plants inoculated

^a^The total rating across multiple plants and replicates

### ALSV-mediated *GmPDS1* silencing in select soybean genotypes was detected in multiple tissue types

**Fig. 6:**
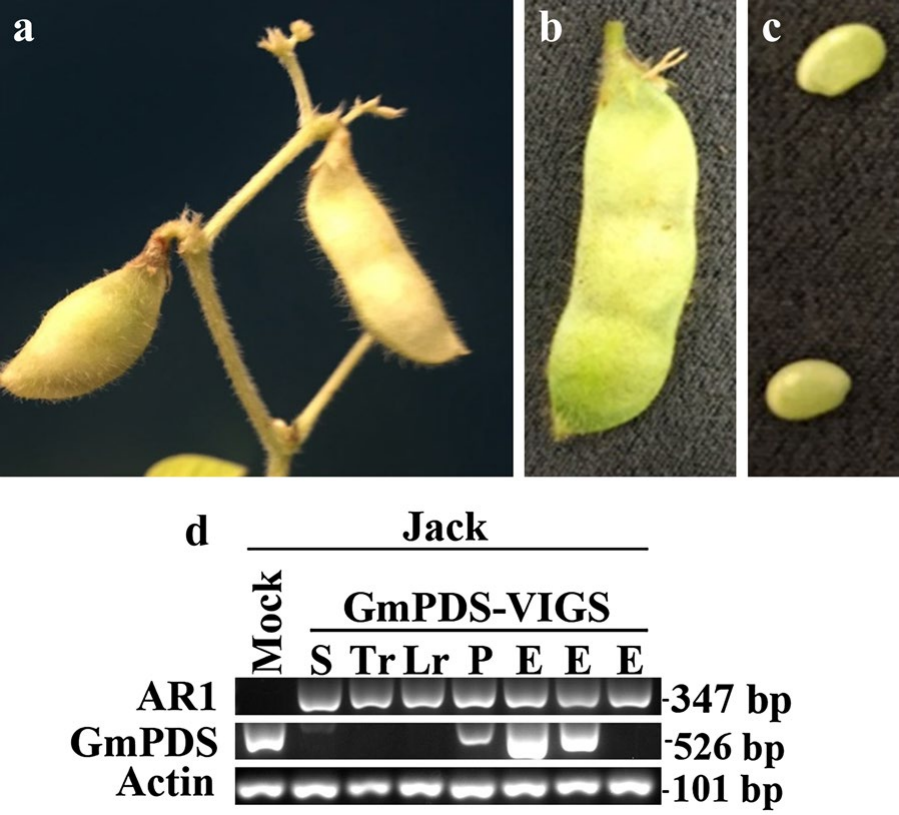
Soybean plants of Jack cultivar inoculated with extracts of *N. benthamiana* leaves that were agro-infiltrated with AR1, AR2-GmPDS, and p19 **a** photo-bleached mature pod, **b** immature pod, **c** young seed. **d** Detection of AR1 and GmPDS1 mRNA levels using semi-quantitative RT-PCR, from different tissues of the cultivar Jack soybean. Mock (inoculation buffer), S (stem), Tr (tap root), Lr (lateral root), P (pod), E (young seed) from three replicates

Finally, a detailed assessment was carried out to monitor *GmPDS1* silencing in different tissue types following rub-inoculation. Roots, stems, pods, and embryos of the cultivar Jack were evaluated for silencing of *GmPDS1*. The cultivar Jack was used for further assessment due to the extensive photo-bleaching observed throughout reproductive stages in previous reports [23]. In the current study, plants that had extensively photo-bleached systemic leaves, AR1 was detected in the roots. Obvious photo-bleaching and AR1 detection was also observed in the stem tissue, mature and immature pods, and embryos of Jack soybean (Fig. 6a–d). Photo-bleached pods and embryos were also noted in PI 567301B indicative of *GmPDS1* silencing. The photo-bleached seeds were propagated to determine the heritability of the silencing phenotype. Although AR1 could be detected in the leaves of germinated Jack and PI 399073 plants, no photo-bleaching symptoms or *GmPDS1* silencing was observed (data not shown).

**Fig. 7:**
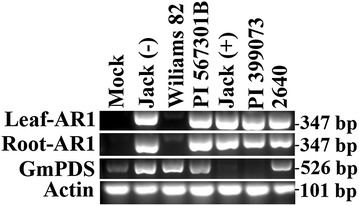
Detection of AR1 in leaves and roots of the same plants with semi-quantitative RT-PCR. The RNA samples were obtained from leaf and root tissues of six soybean genotypes, all of them inoculated with *N. benthamiana* extracts containing AR1, AR2-GmPDS, and p19 Top panel: AR1 in leaf tissue, 2nd panel: AR1 in corresponding root tissue, 3rd panel: *GmPDS1* in root tissue, bottom panel: actin in root tissue. Jack (−) and Jack (+) denote two Jack cultivar individuals with and without photo-bleaching phenotype, respectively. Mock represents Jack cultivar inoculated with inoculation buffer only

A subset of soybean genotypes was selected to test for systemic viral movement to the roots. Genotypes Jack, PI 399073, PI 567301B, RIL 301, Williams 82, and Line 2640 were tested for presence of AR1 in root tissues. Interestingly, in most genotypes AR1 could be detected in root tissue only if *GmPDS1* silencing was observed in the systemic leaves. Conversely, in the 2640 genotype *GmPDS1* silencing was not observed in any individuals, although AR1 was detected in these plants (Fig. 7). Therefore, *GmPDS1* silencing in the leaves may not be a good indicator of viral movement to the roots. These results suggest that ALSV could be suitable for silencing genes that are expressed in roots in a tissue-specific manner.

## Discussion and conclusions

Modification of the ALSV vector allowed for direct, efficient propagation of the VIGS constructs in *N. benthamiana.* Inoculum can be produced and harvested within 5 to 10 dai of *N. benthamiana* infiltration. More importantly the homogenate of agro-infiltrated *N. benthemania* leaves can be directly applied to soybean leaves, thus simplifies and accelerates the VIGS process. By contrast, the particle bombardment procedure used larger quantities of RNA, and involved many more steps, hence introducing more variables. All those demanded more technical skill, which made it difficult to reproduce.

Overall, the newly developed procedure for of the delivery of ALSV VIGS vector is efficient, but also soybean genotype-dependent. One advantage of this procedure is that, because of the limited steps involved, it yielded highly reproducible results between experiments. More importantly, in the genotypes evaluated, ALSV was detected in multiple plant tissues and different developmental stages, suggesting that this vector could have broader usage potential. The production of seeds in *GmPDS1* silenced Jack plants is significant because in previous studies this genotype of soybean did not survive to the seed-bearing stage [23]. This could be due to the modification of the *GmPDS1* insert.

Nine soybean genotypes infected with GmPDS-VIGS displayed photo-bleaching indicative of successful silencing of *GmPDS1*, whereas the other ten genotypes exhibited no *GmPDS1* silencing. Our findings are similar to those of [33] in that, of the multiple soybean germplasm tested for efficiency to *soybean yellow common mosaic virus* based VIGS vector, not all germplasm was susceptible to SYCMV and GmPDS silencing was measured at variable rates between genotypes. Judging from our results and those of [33], more optimization may be needed to gauge the impact of environmental factors such as, growth temperatures and *Agrobacterium* inoculum concentration on the efficiency of VIGS. Apical puncture inoculation only modestly improved silencing efficiency in one soybean genotype. Interestingly, the genotypes showing successful VIGS of *GmPDS1* had similar genetic backgrounds. For instance, PI 567301B had 100% PDS silencing in all plants as well as its progeny RIL 301, which is derived from a cross of Wyandot x PI 567301B. Wyandot, the other parent of RIL301, exhibited no *GmPDS1* silencing, suggesting that resistance to ALSV or ALSV-mediated silencing may be determined by one or a few genetic loci.

Viral infection was detected in plants that exhibited photo-bleaching. However, reduction of *GmPDS1* mRNA levels was variable between genotypes. The genotypes, PI 567301B, RIL 301, PI 399073, and Jack had photo-bleaching on systemic leaves, and GmPDS was detected at varying levels. In root tissue AR1 was detected in PI 567301B, PI 399073, Jack and 2640 even though photo-bleaching wasn’t observed in all genotypes. Visible photo-bleaching of the shoots, pods, and embryos was observed and measured in all genotypes that were susceptible to photo-bleaching in the leaf tissue. Even though ALSV is truly systemic, viral infection apparently did not interfere with phenotypic evaluation. Viral symptoms were restricted to stunting and foliar symptoms including minor leaf crinkling, making this virus a strong VIGS vector candidate for functional analysis studies for some soybean genotypes.

## Additional file

**Additional file 1.** Oligonucleotide sequences used for RT-PCR and semi-quantitative RT-PCR.
