# Supplementary material for: Improved apple latent spherical virus-induced gene silencing in multiple soybean genotypes through direct inoculation of agro-infiltrated Nicotiana benthamiana extract
Source: Plant Methods. 2018 Mar 6;14:19. doi: 10.1186/s13007-018-0286-7 (PMC5838930; doi:10.1186/s13007-018-0286-7)
Supplement: Supplementary file 1 — Additional file 1. Oligonucleotide sequences used for RT-PCR and semi-quantitative RT-PCR. [file 13007_2018_286_MOESM1_ESM.docx]

**Additional File 1.** Oligonucleotide sequences used for RT-PCR and semi-quantitative RT-PCR.

| Target | Oligo name | Sequence |
| --- | --- | --- |
| ALSV-RNA1 | ALSR1-2102F | 5’-CTACTTCTTGGATTGACGCCCT-3’ |
|  | ALSR1-2449R | 5’-GCATATCAAAGAGGGCAGCTCGAGAAAGTGCACTG -3’ |
|  | ALSR1-799F | 5’-CAAGTGAAAACTCGGCGCTCTTTCTC-3’ |
|  | ALSR1-2123R | 5’-AGGGCGTCAATCCAAGAAGTAGTC-3’ |
| ALSV-RNA2 | ALSR2-1364F | 5’-GAGGCACTCCTTATCCTATCAA-3’ |
|  | ALSR2-1523R | 5’-CAAGGTGGTCGTGATTTCACT-3’ |
| *GmPDS1* | GmPDS1-938F | 5’-CCATATGTTGAGGCTCAAGATG-3’ |
|  | GmPDS1-1464R | 5’-AGGTGATCATATGTGTTCTTCAG-3’ |
| *GmACT101* | GmACT101-479F | 5’-TCGTATGAGCAAGGAAATTGG-3’ |
|  | GmACT101-579R | 5’-TAGAGCCACCAATCCAGACAC-3’ |

These primers were used to assess the presence and levels of the AR1 and AR2, and the levels of *GmPDS1*, and the *GmACT101* mRNA, through semi-quantitative RT-PCR. The sequences of *GmACT101* were retrieved from [33].
